# Development of a Human Leukocyte Antigen Score to Predict Progression-Free Survival in Head and Neck Squamous Cell Carcinoma Patients

**DOI:** 10.3389/fonc.2018.00168

**Published:** 2018-05-17

**Authors:** Gunnar Wichmann, Claudia Lehmann, Cindy Herchenhahn, Marlen Kolb, Mathias Hofer, Susanne Wiegand, Andreas Dietz

**Affiliations:** ^1^Clinic for Otorhinolaryngology, Head and Neck Surgery, University Hospital Leipzig, Leipzig, Germany; ^2^LIFE – Leipzig Research Center for Civilization Diseases, University of Leipzig, Leipzig, Germany; ^3^Institute for Transfusion Medicine, University Hospital Leipzig, Leipzig, Germany; ^4^Clinic for Anesthesiology and Intensive Care, University Hospital Leipzig, Leipzig, Germany

**Keywords:** head and neck squamous cell carcinoma, head and neck cancer, human leukocyte antigen, human leukocyte antigen haplotype, progression-free survival, larynx-organ preservation trial, biomarker score, independent predictor

## Abstract

**Background:**

In personalized medicine and treatment stratification of head and neck squamous cell carcinoma (HNSCC), the heterogeneous genetic background of patients is not considered. Human leukocyte antigen (HLA) alleles and HLA haplotypes (HLA traits) are linked to development of HNSCC and affect progression-free survival (PFS) of HNSCC patients but most head and neck oncologists are not familiar with HLA typing. Hence, we developed an HLA-score abstracting from complexity of HLA-typing results to facilitate potential use of HLA-associated hazard ratios (HR) for prognostic stratification.

**Methods:**

The HR for PFS of 8 HLA traits shown to be independent predictors (*Pi*) of PFS in a test cohort (TC) of 90 HNSCC patients were used to build the HLA-score based on the natural logarithm (ln) of the *Pi*-associated HR. Crude ln-transformed HR of the eight *Pi*, alleles B*13 (2), B*35 (1), B*51 (2), DQB1*06 (1), homozygous Cw (1), homozygous DRB4 (2), and haplotypes A*01/B*08 (−6) and B*08/C*07 (4), were summed up to yield the individual patient’s HLA-score. Receiver operating characteristic (ROC) and Kaplan–Meier curves were used to proof the suitability of the HLA-score as prognostic marker for PFS. An independent validation cohort (iVC) of 32 patients treated in the larynx-organ preservation trial DeLOS-II was utilized for validation.

**Results:**

The individual HLA-scores (range −2 to 6) in TC classified HNSCC patients regarding PFS. ROC analysis (area under the curve = 0.750, 95% CI 0.665–0.836; *P* = 0.0000034) demonstrated an optimum cutoff for the HLA-score at 0.5 (97.9% sensitivity, 34.7% specificity), and 70/90 patients in TC with HLA-score > 0 had significant reduced PFS (*P* = 0.001). Applying the same classifier (HLA-score > 0) confirmed these findings in the iVC revealing reduced PFS of 25/32 patients (*P* = 0.040).

**Conclusion:**

HLA traits constitute critical *Pi*. Considering the HLA-score may potentially facilitate the use of genetic information from HLA typing for prognostic stratification, e.g., within clinical trials.

## Introduction

The genetic background of head and neck squamous cell carcinoma (HNSCC) patients and a potential genetic predisposition for development of HNSCC and relapse after successfully applied curative treatment are almost completely ignored. By contrast, somatic mutations, epigenetic changes and divergent gene-expression patterns in HNSCC and tumor-infiltrating lymphocytes present in the tumor are assessed as prognostic and predictive biomarkers (1–8). Main risk factors for carcinogenesis of HNSCC are tobacco and/or alcohol consumption, but the risk is modified by genetic polymorphisms (8–13). In addition, infection with oncogenic subtypes of the human papillomavirus (HPV), especially HPV16 and other high-risk subtypes, is etiologically involved in development of HNSCC of the oropharynx (14). HPV-related HNSCC are characterized by distinct molecular features (2–6, 8, 15). High level tobacco smoking, daily alcohol drinking as well as HPV-related carcinogenesis and especially their simultaneous presence are accompanied by immunotoxicity, genotoxicity, mutagenesis plus increased expression of cytokines and growth factors and exhausted immune response resulting in loss of proliferation control (16). Therefore, and because of the multitude of studies demonstrating the association of these risk factors with HNSCC and outcome, the reasons for developing HNSCC and also relapse after initially curative treatment appear to be obvious. Consequently, and in contrast to many other cancer entities, both occurrence and relapse of HNSCC are mostly seen as attributable to the patient’s lifestyle. However, tobacco smoking and alcohol together explain only 73% of upper aerodigestive tract cancer incidence totally ranging from below 50% in HNSCC-affected women to about 85% in laryngeal and hypopharyngeal HNSCC in men (12). The risk for development of HNSCC is increased by genetic variants in genes encoding enzymes involved in DNA repair or metabolism of alcohol (9–11, 13); Fanconi anemia patients have a 700-fold increased risk for HNSCC and high rate of relapses (17, 18). Moreover, research implicated that polymorphisms in cytokine genes and members of the immunoglobulin supergene family including human leukocyte antigens (HLAs) are involved in either improved or impaired ability to control somatic mutations by adequate immune responses and maintenance of immune surveillance: we recently demonstrated in a German cohort of white Caucasian genetic descent that polymorphisms in HLA, in particular HLA-B antigens and homozygosity in HLA-Cw and DRB4, are associated with increased risk for HNSCC (19). Moreover, the highly frequent disruption of functionally coupled HLA antigens (haplotypes) and presence of uncommon haplotypes in a significant proportion of patients are linked not only to development of HNSCC but even more to reduced progression-free survival (PFS) independent of lifestyle-associated risk factors (19). By contrast, compared with healthy blood donors, some HLA traits are detected in HNSCC in lower frequency. The fewer carriers of such haplotypes have improved PFS, whereas those that are over-represented did not. Hence, genetic heterogeneity seems to account for altered risk of developing HNSCC but also PFS (19). The hazard ratios (HR) for PFS of B*13, B*35, B*51, HLA-DQB1*06, homozygous Cw and DRB4, and the haplotypes A*01/B*08 and B*08/C*07 were stably significant independent predictors (*Pi*) in multivariate analyses (19). They also may be considered as prognostic factors in comparative analyses, e.g., in clinical trials.

However, the use of raw low-resolution tissue-typing results to assess the risk for PFS according to presence of a particular HLA trait and an individual risk attributed to any risk factor including *Pi* appears to be not very useful to estimate the individual patient’s risk for relapse in clinical routine. For the latter purpose, an abstraction from individual polymorphisms could be helpful. Very desirable but not at hand is an easy-to-use way to assess the individual patient’s risk attributable to his/her HLA type. As the aggregation of independent risk factors in a score offers a way to abstract from individual risk factors by summarizing only their (potential) impact as *Pi* (20), we had the hypothesis that HR for PFS may be useful to construct an HLA-score. Therefore, we newly defined an HLA-score based on published HR from our recent findings in a test cohort (TC) (19). Here, we aim to verify a potential impact of the scored HLA traits on PFS of HNSCC and to particularly elucidate, if this HLA-score reliably predicts outcome differences in the context of clinical trials. Hence, low-resolution HLA typing of leukocytes from an independent validation cohort (iVC), 32 laryngeal/hypopharyngeal HNSCC patients treated in the DeLOS-II larynx-organ preservation trial (20, 21), was performed. Related to their HLA-scoring the PFS in the iVC was analyzed and confirmed the prognostic value of the HLA-score.

## Materials and Methods

### HNSCC Patients and Study Population

This study was carried out in accordance with the recommendations of the guidelines of the ethics committee of the Medical Faculty of the University Leipzig. The protocol was approved by the ethics committee of the Medical Faculty of the University Leipzig (vote no. 201-10-12072010 and no. 202-10-12072010). All subjects gave written informed consent in accordance with the Declaration of Helsinki.

#### Test Cohort

Blood samples were from histopathologic confirmed HNSCC patients (Table 1) of white Caucasian genetic descent diagnosed and treated between 08/2010 and 05/2011 at the ENT Department of the University Hospital Leipzig. 12 of the 90 patients in the TC were treated in the larynx-organ preservation trial DeLOS-II (21) (NCT00508664; advanced HNSCC of the hypopharynx or larynx receiving induction chemotherapy followed by radiotherapy ± cetuximab; *n* = 12). For HLA typing, genomic DNA was isolated using the salting out procedure (22) from leukocytes of blood samples. Low-resolution DNA-typing was performed using PCR-SSP for HLA-A, B, Cw, HLA-DRB1, DRB3/4/5, and DQB1 as described elsewhere (18).

**Table 1:** Main characteristics of the head and neck squamous cell carcinoma patients of the test cohort (TC; *N* = 90) and independent validation cohort (iVC; *N* = 32) investigated.

		TC		iVC		
				
Covariate	Category	*n*	(%)	*n*	(%)	*P* value
Sex	Female	12	(13.3)	5	(15.6)	0.748
	Male	78	(86.7)	27	(84.4)	

Localization	Oropharynx	28	(31.1)	0	(0.0)	<0.001
	Other	62	(68.9)	32	(100.0)	
	Oropharynx	28	(31.1)	0	(0.0)	<0.001
	Hypopharynx	20	(22.2)	19	(59.4)	
	Larynx	24	(26.7)	13	(40.6)	
	Other	18	(20.0)	0	(0.0)	

T category	Tx	1	(1.1)	0	(0.0)	0.025
	T1	13	(14.4)	0	(0.0)	
	T2	21	(23.3)	3	(9.4)	
	T3	21	(23.3)	15	(46.9)	
	T4a	32	(35.6)	14	(43.8)	
	T4b	2	(2.2)	0	(0.0)	

N category	N0	32	(35.6)	3	(9.4)	0.010
	N1	7	(7.8)	1	(3.1)	
	N2a	5	(5.6)	0	(0.0)	
	N2b	22	(24.4)	13	(40.6)	
	N2c	9	(10.0)	14	(43.8)	
	N3	5	(5.6)	1	(3.1)	

N category	N0	32	(35.6)	3	(9.4)	0.005
	N+	58	(64.4)	29	(90.6)	

M category	M0	87	(96.7)	32	(100.0)	
	M1	3	(3.3)	0	(0.0)	

Stage	UICC I	8	(8.9)	0	(0.0)	0.055
	UICC II	11	(12.2)	0	(0.0)	
	UICC III	9	(10.0)	4	(12.5)	
	UICC IVA	53	(58.9)	27	(84.4)	
	UICC IVB	6	(6.7)	1	(3.1)	
	UICC IVC	3	(3.3)	0	(0.0)	

Stage	Early	19	(21.1)	0	(0.0)	0.006
	Advanced	71	(78.9)	32	(100.0)	

Human papillomavirus (HPV) status	High-risk HPV-DNA+	17	(18.9)	0	(0.0)	0.008
	High-risk HPV-DNA−	73	32	(100.0)	
	HPV16-DNA+	13	(14.4)	0	(0.0)	0.023
	HPV16-DNA−	77	(85.6)	32	(100.0)	
	HPV16-DNA + RNA+	8	(8.9)	0	(0.0)	0.081
	HPV16 RNA−	82	(91.1)	32	(100.0)	

Tobacco smoking behavior	Non-smoker	19	(21.1)	0	(0.0)	0.004
	Smoker	70	(77.8)	32	(100.0)	
	Missing	1	(1.1)	0	(0.0)	
	Non-smoker	19	(21.1)	0	(0.0)	0.046
	<10 pack years	2	(2.2)	2	(6.3)	
	10 < 20 pack years	5	(5.6)	5	(15.6)	
	20 < 30 pack years	8	(8.9)	4	(12.5)	
	30 < 40 pack years	26	(28.9)	9	(28.1)	
	≥40 pack years	29	(32.2)	12	(37.5)	
	Missing	1	(1.1)	0	(0.0)	

Alcohol consumption	No	9	(10.0)	1	(3.4)	0.218
	Yes	80	(88.9)	31	(96.6)	
	Missing	1	(1.1)	0	(0.0)	

Alcohol consumption category	0	10	(11.1)	1	(3.4)	0.455
	>0 < 30 g/day	31	(34.4)	11	(34.4)	
	30 < 60 g/day	24	(26.7)	8	(25.0)	
	≥60 g/day	24	(26.7)	12	(37.5)	
	Missing	1	(1.1)	0	(0.0)	

Therapy	Surgery (Op)	19	(21.1)	0	(0.0)	<0.001
	Radiotherapy (RT)	6	(6.7)	0	(0.0)	
	Op + PORT	19	(21.1)	0	(0.0)	
	Op + PORChT	16	(17.8)	0	(0.0)	
	Primary concurrent RChT	3	(3.3)	0	(0.0)	
	DeLOS-II (IC + RT)	12	(13.3)	32	(100.0)	
	IC + Op + PORT	13	(14.4)	0	(0.0)	
	Best supportive care (BSC)	2	(2.2)	0	(0.0)	

Therapies applied	Monomodal	25	(27.8)	0	(0.0)	0.002
	Multimodal	63	(70.0)	32	(100.0)	
	BSC	2	(2.2)	0	(0.0)	
	Received Op	68	(75.6)	5	(15.6)	<0.001
	No	22	(24.4)	27	(84.4)	
	Received RT	69	(76.7)	32	(100.0)	0.003
	No	21	(23.3)	0	(0.0)	
	Received chemotherapy (ChT)	44	(48.9)	32	(100.0)	<0.001
	No	46	(51.1)	0	(0.0)	
	Received Op + RT	49	(54.4)	5	(15.6)	<0.001
	No	41	(45.6)	27	(84.4)	
	Received ChT + RT	44	(48.9)	32	(100.0)	<0.001
	No	46	(51.1)	0	(0.0)	
	Received Op + ChT + RT	30	(33.3)	5	(15.6)	0.057
	No	60	(66.7)	27	(84.4)	

#### Independent Validation Cohort

DNA samples from peripheral blood of additional 32 of the 52 LHSCC patients treated in the DeLOS-II trial in Leipzig (20, 21) underwent low-resolution HLA typing utilizing BMT OneLambda SSO-typing kits for HLA-A, B, Cw, DQ, and DR according to the manufacturer’s instructions. Blood samples of eight DeLOS-II patients were not available as three patients were already deceased, two patients were reluctant to participate in the study and donate blood for genotyping, and three patients were lost to follow-up. Avoiding overlap with the 12 DeLOS-II patients in TC, the iVC consists of *N* = 32 independent samples. Presence of the eight HLA traits constituting *Pi* of PFS in the TC was assessed as described (19).

### Development of the HLA-Score

The recently published HR of HLA traits detected in the TC that consistently remained significant *Pi* for PFS after bootstrapping with 1,000 iterations (19) were used to build the HLA-score. This data set from the TC (19) fulfils all prerequisites to classify the patients based on a score combining up to eight independent predictors (*n* = 90 > 64 = 8^2^). The HR for each of the eight HLA traits was transformed into its natural logarithm, ln HR (Table 2). Absence of a genetic predictor scored 0, while its presence scored with the crude ln HR denoted in brackets: HLA-B*13 (2), HLA-B*35 (1), HLA-B*51 (2), HLA-DQB1*06 (1), homozygous HLA-Cw (1), homozygous HLA-DRB4 (2), haplotype A*01/B*08 (−6), haplotype B*08/C*07 (4). These scores were summed up to build the HLA-score of the individual patient.

**Table 2:** Significant independent predictors for progression-free survival of head and neck squamous cell carcinoma patients according to published data from the multivariate Cox regression model (18) used to define the human leukocyte antigen (HLA) score.

Covariates	TC					Validation cohort			
		
	HR	(95% CI)	*P* value (2-sided)	*P* value (2-sided; in bootstrapping)^a^	*n* (%)	*n* (%)	*P* value (2-sided)^b^	ln HR	HLA-score
HLA-B*13	7.460	(2.212–25.16)	0.0012	0.025	11 (12.2)	4 (12.5)	0.967	2.010	**2**
HLA-B*35	2.630	(1.543–4.485)	0.0004	0.017	15 (16.7)	5 (15.6)	0.891	0.967	**1**
HLA-B*51	9.278	(2.270–37.92)	0.0019	0.015	8 (8.9)	4 (12.5)	0.556	2.228	**2**
HLA-DQB1*06	1.890	(1.152–3.101)	0.0117	0.037	40 (44.4)	10 (31.3)	0.192	0.637	**1**
Homozygous HLA-Cw	4.292	(1.864–9.888)	0.0006	0.001	27 (30.0)	10 (31.3)	0.895	1.457	**1**
Homozygous HLA-DRB4	9.513	(2.787–32.47)	0.0003	0.007	10 (11.1)	7 (21.9)	0.131	2.253	**2**
Haplotype A*01/B*08	0.003	(0.000–0.054)	0.000056	0.003	11 (12.2)	3 (9.4)	0.664	−5.809	**−6**
Haplotype B*08/C*07	74.856	(12.58–445.4)	0.000002	0.001	16 (17.8)	5 (15.6)	0.782	4.316	**4**

*Hazard ratio (HR plus 95% CI in brackets) and the associated 2-sided *P* value, plus *P* values from bootstrapping applying 1,000 iterations are shown accompanied by their frequencies in the test cohort (TC) (*N* = 90) and independent validation cohort (*N* = 32)*.

*The HLA-scores (highlighted bold) for the individual respective HLA trait are derived from the natural logarithm (ln)-transformed HR*.

*^a^Bootstrapping using 1,000 iterations*.

*^b^Comparison of frequencies in TC versus iVC using Pearson’s χ^2^ test*.

### Statistical Analysis

Pearson’s chi-square (χ^2^) test for contingency tables was used to analyze differences between groups. PFS was chosen as end point in survival analyses and measured from registration date until date of either cancer-related death or relapse, censoring patients without any malignancy at last follow-up, or deaths not related to cancer. As relapse were considered local recurrence, lymph node or distant metastasis, or second primaries. PFS was analyzed using Kaplan–Meier curves (SPSS version 20, IBM Corporation, Armonk, NY, USA) applying log-rank tests (Mantel–Cox). Receiver operating characteristics (ROC) for PFS versus HLA-score were assessed using SPSS. *P* values < 0.05 from two-sided tests were considered significant.

## Results

Table 1 shows the characteristics of both cohorts. Some significant inequalities in risk-factor distributions were detected. According to the study protocol of the DeLOS-II trial all 32 LHSCC patients of the iVC were of advanced stage (UICC III, IV) with higher T and N categories. They were exclusively smokers and not HPV-related (*P* < 0.01), and based on the per-protocol treatment in DeLOS-II (20, 21) their treatment differed significantly from the TC (Table 1). Despite the lower case number in iVC, a comparable distribution and frequency of the HLA traits included in the HLA-score was observed and found being without any significant difference (Table 2).

The individual patient’s HLA-score as defined by the sum of the crude ln-transformed HR of the eight HLA traits resulted in a comparable distribution of HLA-scores (*P* = 0.683). These scores were found in cohorts TC/iVC: −2 (*n* = 3/2; 3.3/6.3%), −1 (*n* = 5/1; 5.6/3.1%), 0 (*n* = 12/4; 13.3/12.5%), 1 (*n* = 29/6; 32.2/18.8%), 2 (*n* = 22/11; 24.4/34.4%), 3 (*n* = 9/4; 10/12.5%), 4 (*n* = 4/0; 4.4/0%), 5 (*n* = 3/2; 3.3/6.3%), and 6 (*n* = 3/2; 3.3/6.3%).

**Figure 1:**
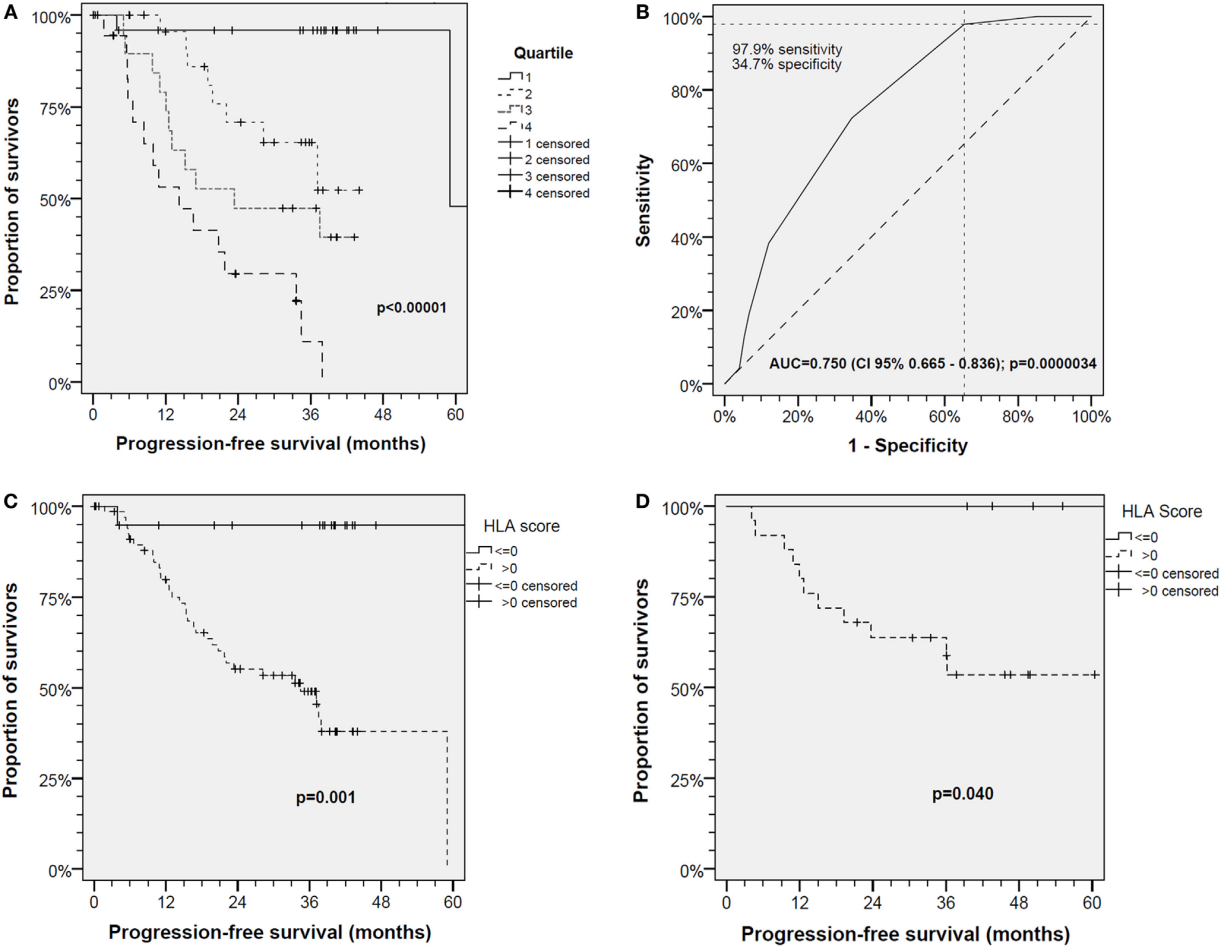
The human leukocyte antigen (HLA)-score is an independent predictor of progression-free survival (PFS) in head and neck squamous cell carcinoma (HNSCC) patients. **(A)** PFS in the *N* = 90 HNSCC patients of the test cohort (TC) respective to HLA-score quartiles; **(B)** receiver operating characteristics for PFS event versus HLA-score demonstrate high significance and optimum classification with 97.9% sensitivity and 34.7% specificity at a cutoff of 0.5 in the TC; **(C)** PFS in the TC of *N* = 90 HNSCC patients classified according to cutoff 0.5; **(D)** PFS in the *N* = 32 patients of the independent validation cohort classified according to cutoff 0.5 confirms the findings in the TC.

Figure 1A shows Kaplan–Meier analyses of PFS in the TC according to HLA-score quartiles. The PFS of HNSCC patients is inversely correlated with HLA-score quartiles. Applying the log-rank test, a significant different PFS of patients of the TC was observed (*P* < 0.00001).

Receiver operating characteristic analyses revealed a significant area under the curve (AUC) for PFS event versus HLA-score (AUC = 0.750, 95% CI 0.665–0.836; *P* = 0.0000034) with HLA-score 0.5 being the optimum cutoff for discrimination of HNSCC patients with good *versus* impaired PFS in the TC (Figure 1B). Binary classification of TC patients applying this cutoff offers 34.7% specificity and 97.9% sensitivity corresponding to a negative predictive value (NPV) of 94.7% of the HLA-score ≤ 0 for relapse or cancer-related death.

Kaplan–Meier analyses confirmed the optimal binary classification into groups of patients without HLA-attributable risk (HLA-score ≤ 0; *n* = 20) versus those at risk (HLA-score > 0; *n* = 70) and achieved in the TC the most significant discrimination between groups with deviating PFS (*P* < 0.001; Figure 1C).

The impact of the HLA-score on PFS was further analyzed in the iVC. The HLA-score ≤ 0.5 had in this cohort an NPV of 100%. In full agreement, and despite the smaller sample size of *N* = 32, the HLA-score ≤ 0 and >0 exactly predicted in Kaplan–Meier curves either prolonged or shortened PFS, respectively, of the iVC patients (*P* = 0.040; Figure 1D).

## Discussion

The TC of 90 HNSCC patients demonstrated altered frequencies of HLA antigens and two-locus haplotypes, as well as high frequent homozygosis in Cw and DRB4. The eight HLA traits identified as stable *Pi* respective to a significant impact on PFS of HNSCC patients can be combined to build an HLA-score. As shown here for the first time, the HLA-score of an HNSCC patient, which is the sum of crude ln-transformed HR of the eight HLA traits, is inversely correlated with the PFS and is a *Pi*. Multivariate analysis in the TC revealed significant altered PFS in carriers of homozygous Cw and DRB4, four HLA-B alleles, and two haplotypes (19). The in Wichmann et al., 2017 (19) applied Cox model (due to inclusion of HLA traits) no longer included N category N0 versus N+ (*P* = 0.520), alcohol consumption (*P* = 0.541), sex (*P* = 0.118), and age at diagnosis (*P* = 0.253), as these covariates lowered its overall significance (19). The here newly established HLA-score defines groups of HNSCC patients with significant different PFS independent from these “classical” risk factors for HNSCC (19). This may be seen also in the context of unexplained risk for development of HNSCC outside the main risk factors tobacco and alcohol (12).

The cutoff 0.5 allowed for discrimination of patient groups with different PFS according to their HLA-score also in the iVC. Patients with HLA-score > 0 had a significant higher risk for relapse (*P* = 0.040) compared with patients with HLA-score ≤ 0 who were without event and confirmed the findings in the TC (Figure 1D). This suggests that the HLA-score is potentially able to summarize the HR of HLA traits in a single measure that inversely correlates with the PFS and may be useful as stratification factor for clinical trials, observational studies or in personalized medicine.

Taken together, the results obtained by applying the HLA-score demonstrate the possibility that HLA traits are able to explain at least partially the high level of variance in outcome within clinical trials as demonstrated by the 32 patients of the iVC (Figure 1D) treated in the DeLOS-II larynx-organ preservation trial (20, 21). The distribution of HLA traits between study arms therefore may affect the outcome in clinical trials. Frequencies of antigens and haplotypes shown to be *Pi* in the TC range between a few percent (4.4 up to 23.9%), and homozygosity in Cw and DRB4 was detected in 30.0 and 11.1%, respectively (19). As effective randomization regarding multiple risk factors each of them individually present in low frequency requires prohibitive high case numbers, unevenly distributed HLA traits could explain failure or irreproducibility of clinical trials even if higher case numbers are compared. This will occur as long as the multitude of low frequent *Pi* is not considered in stratification before randomization. The HLA-score may allow for overcoming this issue by assessment of the risk associated with particular HLA traits which are *Pi*.

What are the reasons behind the effect of HLA traits and the HLA-score on PFS of HNSCC? In HNSCC oncology, the exposure to tobacco smoke and alcohol are the dominant and most accepted risk factors for development and relapse of HNSCC as they are observed in high frequency and shown to be causative for mutations, e.g., in oncogenes or inactivation of tumor-suppressor genes but also resistance to immune surveillance (23). Research showed a broad spectrum of mutated genes and affected signaling pathways in HNSCC (2–5, 8). However, most sporadic somatic mutations or viral infections potentially causing neoplastic transformation are controlled by the immune system. Consequently, altered peptides derived from mutated or viral proteins are in majority efficiently presented to CTL (CD8^+^ cytotoxic T lymphocytes) *via* HLA-A, B, and Cw enabling antigen-specific CTL to bind and delete cancerous cells expressing aberrant or viral proteins. Obviously, these mechanisms work well in most people but not so well in most HNSCC patients. This may be caused either by inadequate binding of T cell receptors to MHC:peptide complexes or incapability of the HLA-proteins to process tumor-associated antigens (TAA) by proteolytic cleavage and to bind particular TTA-derived peptides. Besides the often observed MHC class I loss in HNSCC (24, 25) allowing immune escape, there are indeed huge differences between certain HLA antigens to bind TAA-derived peptides (26). HLA antigens combined in particular haplotypes may have gaps in the capability to bind and present particular TAA-derived peptides. Consequently, the HLA antigens and haplotypes differ in the probability to appropriately activate T cells and get rid of cancer cells expressing peculiar proteins (27). Such impaired competence to maintain immune surveillance is suggested to be related to particular HLA alleles (and haplotype combinations) to efficiently bind and present altered peptides (26) and trigger deletion of the mutated cell by CTL (27). This may cause varying numbers of tumor-infiltrating CD8^+^ T cells within HNSCC as shown recently (5, 8, 28).

The HLA-score reliably predicts PFS of HNSCC patients. Even without any clinical information, the stratification using the HLA-score distinguished HNSCC with significant deviating PFS in TC and iVC (Figure 1). We detected significant superior outcome in patients with HLA-score ≤ 0 with strongly improved relative risk and odds ratios by optimum classification identified in ROC analyses (Figure 1B). It might be important that the HLA-score is able to indicate significant outcome differences even within the small sample of 32 patients in the iVC which moreover had a huge heterogeneity in many response-associated parameters (20). This is even more important as the case number of 32 is much too low to demonstrate significant outcome differences between LHSCC patients related to the treatment, e.g., arm A versus B (20).

Within our study, we noticed that sole presence of one of the eight *Pi* constituting the HLA-score not necessarily predicts the outcome regarding reduced PFS. This is in full agreement with the general explanations by Powers (29) who stated that the directions of implications are not in general dependent: if *Pi* is one of several independent possible causes of the condition *R* (i.e., PFS), *Pi* → *R* is strong, but *R* → *Pi* is in general weak for any specific *Pi*. If *Pi* is one of several contributing factors to the condition *R, Pi* → *R* is weak for any single *Pi*, but *R* → *Pi* is strong (29). Regarding our HLA-score, this means that each of the eight included independent predictors *Pi* for the condition *R* (event regarding PFS) alone is weak in explaining the outcome (PFS), and presence of a particular *Pi* (either alleles HLA-B*13, B*35, B*51, DQB1*06, haplotypes B*08/Cw*07 and A*01/B*08, homozygous DRB4 or Cw) stands not against good outcome in general. *Vice versa*, the HLA-score ≤ 0 summarizing the eight *Pi* strongly predicts superior outcome, and the outcome explains the high predictive value of the HLA-score (Figure 1).

Our study has some limitations. The impossibility of familial HLA typing due to unavailability of DNA from parents of our patients allowed only for evaluation of estimated haplotypes (phenotypic combinations). However, this is the appropriate and most-often used method for analysis of HLA haplotypes and disease associations (30–32). The small sample size in the iVC might have caused reduced power to replicate the findings in the TC regarding particular HLA traits which are present in frequencies below 10%, e.g., HLA-B alleles and estimated haplotypes, on PFS. However, the abovementioned causality according to *Pi* → *R* applies (29).

Nevertheless, the recently detected HLA-trait dependence of PFS and the possibility to use the newly developed HLA-score requests further investigations to provide proof of reproducibility within cohorts of different genetic background. The possibility exists that the HLA-score can apply only to patients of white Caucasian genetic descent as particular alleles/antigens may behave in a different way when present in a different genetic environment. Some similarities may exist (e.g., the role of homozygosity in particular of Cw respective to an impaired prognosis), but ethnicity-dependent differences in distribution of particular HLA antigens/alleles and the association of varying haplotypes, e.g., with autoimmune diseases suggest the existence of such inequalities. They might have been at least partially responsible for unresolved issues of varying outcome especially observed in multinational clinical trials. For instance, in the SPECTRUM trial a huge difference was seen outcome of patients treated in Europe and in the U.S. of America compared with patients from the Asian-Pacific region (33) which we expected being at least partially related to genetic heterogeneity in HLA traits of the ethnicities. Therefore, it would be very welcome if HLA typing at least at the low-resolution level is performed within clinical trials including HNSCC patients of other ethnicities to elucidate HLA traits with the potential to differently affect outcome. This should clarify if patient stratification according to HLA traits is possible in multinational trials and if the here presented HLA-score is able to improve reproducibility in future clinical trials.

The identification of a subgroup of patients based on the HLA-score in both cohorts with uniquely superior PFS argues for consideration of HLA traits as stratification factors in head and neck oncology.

## Data Availability Statement

The datasets analyzed for this study are available on request from GW, gunnar.wichmann@medizin.uni-leipzig.de.

## Ethics Statement

This study was carried out in accordance with the recommendations of the guidelines of the ethics committee of the Medical Faculty of the University Leipzig. The protocol was approved by the ethics committee of the Medical Faculty of the University Leipzig (vote no. 201-10-12072010 and no. 202-10-12072010). All subjects gave written informed consent in accordance with the Declaration of Helsinki.

## Author Contributions

GW designed and coordinated the study. CH, CL, SW, MH, AD, and GW sampled biological specimen from HNSCC patients. Clinical data were provided by SW, MH, AD, and GW. CH, CL, and GW performed HLA typing. CH and GW assessed antigen and haplotype distribution. GW performed statistical analyses and developed the HLA-score. MK and GW discussed statistical models and interpreted the data. GW wrote the first version of the paper. All the authors approved the report.

## Conflict of Interest Statement

The authors declare that the research was conducted in the absence of any commercial or financial relationships that could be construed as a potential conflict of interest.
